# Machine Learning on Prediction of Relative Physical Activity Intensity Using Medical Radar Sensor and 3D Accelerometer

**DOI:** 10.3390/s23073595

**Published:** 2023-03-30

**Authors:** Attila Biró, Sándor Miklós Szilágyi, László Szilágyi, Jaime Martín-Martín, Antonio Ignacio Cuesta-Vargas

**Affiliations:** 1 Department of Physiotherapy, University of Malaga, 29071 Malaga, Spain; abiro@uma.es (A.B.);; 2 Department of Electrical Engineering and Information Technology, George Emil Palade University of Medicine, Pharmacy, Science, and Technology of Targu Mures, Str. Nicolae Iorga, Nr. 1, 540088 Targu Mures, Romania; 3 Biomedical Research Institute of Malaga (IBIMA), 29590 Malaga, Spain; 4 Computational Intelligence Research Group, Sapientia Hungarian University of Transylvania, 540485 Targu Mures, Romania; 5 Physiological Controls Research Center, Óbuda University, 1034 Budapest, Hungary; 6 Legal and Forensic Medicine Area, Department of Human Anatomy, Legal Medicine and History of Science, Faculty of Medicine, University of Malaga, 29071 Malaga, Spain; 7 Faculty of Health Science, School of Clinical Science, Queensland University Technology, Brisbane 4000, Australia

**Keywords:** medical radar, session rating of perceived exertion, distant sensing in sports, fatigue control, machine learning, assessment

## Abstract

Background: One of the most critical topics in sports safety today is the reduction in injury risks through controlled fatigue using non-invasive athlete monitoring. Due to the risk of injuries, it is prohibited to use accelerometer-based smart trackers, activity measurement bracelets, and smart watches for recording health parameters during performance sports activities. This study analyzes the synergy feasibility of medical radar sensors and tri-axial acceleration sensor data to predict physical activity key performance indexes in performance sports by using machine learning (ML). The novelty of this method is that it uses a 24 GHz Doppler radar sensor to detect vital signs such as the heartbeat and breathing without touching the person and to predict the intensity of physical activity, combined with the acceleration data from 3D accelerometers. Methods: This study is based on the data collected from professional athletes and freely available datasets created for research purposes. A combination of sensor data management was used: a medical radar sensor with no-contact remote sensing to measure the heart rate (HR) and 3D acceleration to measure the velocity of the activity. Various advanced ML methods and models were employed on the top of sensors to analyze the vital parameters and predict the health activity key performance indexes. three-axial acceleration, heart rate data, age, as well as activity level variances. Results: The ML models recognized the physical activity intensity and estimated the energy expenditure on a realistic level. Leave-one-out (LOO) cross-validation (CV), as well as out-of-sample testing (OST) methods, have been used to evaluate the level of accuracy in activity intensity prediction. The energy expenditure prediction with three-axial accelerometer sensors by using linear regression provided 97–99% accuracy on selected sports (cycling, running, and soccer). The ML-based RPE results using medical radar sensors on a time-series heart rate (HR) dataset varied between 90 and 96% accuracy. The expected level of accuracy was examined with different models. The average accuracy for all the models (RPE and METs) and setups was higher than 90%. Conclusions: The ML models that classify the rating of the perceived exertion and the metabolic equivalent of tasks perform consistently.

## 1. Introduction

The use of body sensors in sports (such as wearable body sensors, head impact sensors, mouth guards, heart rate (HR), and motion sensors) to measure human performance and behavior in wellness and healthcare applications is fast becoming a reality [1,2,3]. Moreover, various novel sensing approaches, such as textile-based wearable sensors, microneedles, microfluidic sensor platforms, and flexible sensor form factors, to accurately measure human performance in sports (and other contexts), have been recently introduced [4].

**Figure 1:**
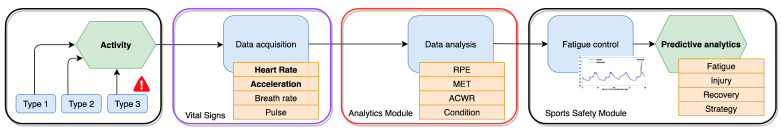
Next-Generation Sports Safety Approach.

These technologies can adequately monitor and measure the physical performance of an athlete. Consequently, they can be used to continuously collect biomechanical and physiological performance data to prevent significant adverse effects (sometimes even death) and increase the team’s efficiency by maintaining the players in a virtually optimal physical condition. In active indoor sports [5] games such as ice hockey, handball, and basketball, the players may be more or less tired, exhausted, and losing water (causing dehydration [6,7], which is also a cause of hypernatremia) depending on the game velocity and intensity level, as well as the surrounding ecosystem, including humidity/temperature conditions [8]. In addition, players’ performance fluctuates from game to game based on the time, location, and field position. Recent advances in next-generation technologies enable intelligent “health” sensors, dedicated radar sensing devices, three-axial accelerometer sensors, and various health activity and health-state tracking pathways to achieve these objectives [8]. Figure 1 shows the next-generation (expected) approach of sports safety, as a novelty concept, where Type 1 activities are the sports which allow any type of sensors during game sessions, Type 2 are the injury-based regulated and limited ones, and Type 3 which does not allow any wearable or attached sensors, just remote sensing. The novelty consists not only of the early adoption of a wearable with distant sensing solutions, such as medical radar sensors, but in the introduction of artificial intelligence as main data analytics and sports safety assets.

The most challenging aspect of indoor performance sports is how to achieve data collection in an invisible but protective way from athletes using well-known devices and sensing networks [8] (e.g., medical health sensors, smart watches, or various types of activity sensors) to provide timely updates to key stakeholders (including managers, trainers, doctors, and coaches) regarding athlete performance and the possibility of them entering dangerous health states (such as excessive hydration or dehydration). The data’s hidden patterns [9] will be identified using real-time predictive algorithms based on incoming sensor data streams [10]. The initial stage is identifying the least harmful device and communication route and setting up the data-collecting pipeline.

Controlling tiredness [11] through non-invasive athlete monitoring is one of the most pertinent subjects in sports safety today. It enhances injury prevention [12] by decreasing injury risks thanks to fatigue control. There is a risk of contact-related harm when accelerometer-based smart trackers, activity measurement wristbands, or smart watches are used to measure health parameters during high-performance sports activities; therefore, these devices should not be utilized.

In contrast to other approaches, radar sensors use a 24 GHz Doppler radar sensor [13,14,15] to detect vital signs, such as the heart rate (HR) and respiration. This is accomplished without touching or approaching the subject. Therefore, it can forecast the degree of activity that will be performed. Three-axial (3D) accelerometer [16] sensors are used as dedicated devices attached to protectors (with potential adaptability to ice hockey and American football) or used as wristed devices, such as smart watches.

## 2. Objectives

With the rise of mHealth applications in the leisure sports sector, connected smart wearables (from wristbands to t-shirts that manage movement) record our steps and provide valuable, vital data. During this transition, the unimaginable has become a reality, altering our lifestyle, the sport we practice, and sport itself (e.g., how to practice more effective fitness). Currently, everyday exercise is fashionable. Many of those who participate in sports or controlled training sessions have already incorporated IoT fitness gadgets or various activity trackers into their lives, the primary function of which is to aid athletes in their practice.

Performance sports, especially indoor ones, have limitations because of injury prevention. Combining the radar sensors and accelerometers—conducting an invisible data acquisition pipeline—during sports activities will make it possible to maintain a safer sports ecosystem. The main conclusion of this state-of-the-art study is as follows: nowadays, no vitals systems primarily oriented to athletes have yet been integrated to gather data using radar sensors or in an invisible way, i.e., in a contactless form. The raw health data, without further modification, could give us more reliable information about perceived exertion or fatigue. These will help coaches prepare complex models using artificial intelligence to maximize the athletes’ capabilities.

This study firstly examines the viability and limitations of integrating machine learning (ML) algorithms with medical radar sensors, and then of combining the synergy of three-dimensional acceleration sensor data to assess the intensity of elite athletes’ relative physical activity [17]. The objectives of this research are:

1. To confirm the feasibility of the adaptability of medical radar sensor technology [18] into sports, highlighting at the same time the limitations of the technology.
2. To leverage the latest advances in wearable and radar sensor technology, combined with mobile computing platforms, to deliver an athlete monitoring solution to critical stakeholders interested in maximizing athlete safety and performance.
3. To add objective alternatives to the measure of the rating of perceived exertion (RPE) [19] on Borg’s RPE scale [20,21,22] as a control option for sports safety.
4. To research the adaptability of ML models into sports safety, by implementing RPE or the session rating of perceived exertion (sRPE) [23], METs prediction prototypes from heart rate (HR), and three-axial acceleration information, using the latest research relevant to MET sports, gathering critical data and insights for further research with big data.

The efficacy of ML models such as the random forest, Xtreme gradient boosting, and decision tree, as well as regressions such as Ordinary Least Squares (OLS), Least Absolute Shrinkage and Selection Operator (LASSO), and methods with pre-made feature sets were used to predict the relative level of physical activity and energy expenditure.

## 3. Materials and Methods

The first phase of this research validates the medical radar sensors’ usability in the sports sector; the real-time remote data acquisition process [24] and the initial feasibility validation experiments were conducted based on that. In the validation phase, the research participants were professional athletes, with a minimum of 30 activities per player sample of participants. During the experiments, we used a fixed installation, by attaching the radar sensors to benches, resulting in an optimal measurement range distance of 30–50 cm between athletes and sensor device. These samples were used to validate the usability of medical radar sensors in the sports domain. For a deeper analysis, it is suggested to perform a deeper analysis per sport, such as dedicated trials, as dedicated trials.

### 3.1. Data

After the evaluation of the results, we moved to the second phase of experiments and we conducted the experiments using an already validated dataset with combined information about heart rate and the 3-axial acceleration to determine the feasibility of ML algorithms by using the PAMAP2 DATASET [25,26]. The “PAMAP2 Physical Activity Monitoring” (PAMAP2) dataset contains around 18 activities (e.g., walking, running, cycling, Nordic walking, football, rope jumping, etc.). Its most important attributes are the following: (a) has multivariable, time-series data characteristics, (b) 3,850,505 instances, (c) 52 attributes which are the following: the manual data have been prepared to conform to the General Data Protection Regulation (GDPR) by using an anonymized dataset [25,26,27]. Table 1 shows the anonymized information about the users of our experiments from PAMAP2, providing information about gender, age, height, weight, resting HR, maximum HR (calculated value), and dominant hand.

For the ML algorithm feasibility test, we used synthetic generated datasets and anonymized sample datasets [28]. It has collected the minimum data threshold for a specific data science project. The data adjustments were conducted with dedicated Python scripts. For analysis, we used Google Colab Platform [24].

### 3.2. Experimental Environment

The modeling and AI experiments were run on the following configuration: MBO Gigabyte Z390 Aorus Pro, CPU INTEL Core i7-8700K 3.7 GHz 12 MB LGA1151, DDR4 32 GB 3600 MHz Kingston HyperX Predator Black CL17 KIT2, VGA MSI RTX 2080 Ventus 8 GB, SSD M.2 SAMSUNG 970 Pro 1 TB, Corsair RMx (2018) 750 W Modular 80+ Gold, as well as dedicated notebooks on the Google Colab platform [24].

Medical Radar Sensor: MIO MS106 B100 with WM7RAZ01. A 24 GHz band microwave Doppler sensor, sensing range: 4 m × 4 m × 2.5 m, optimal measurements: within 30–50 cm from body, DC 5 V, 0.4 A. Monitoring functions: heartbeat, breath rate, body movements. Breath detection: 10 cm–2.5 m. Communication options of this radar sensor family: USB, Bluetooth, Wifi, Zigbee.

Three-Dimensional Accelerometer: In our research, we used ultralow-power high-performance three-axis linear accelerometer (Discovery Mini) with internal eeprom memory, having as notification service the buzzer or vibration features. The device was installed with a 400 mAh battery. The device has two operating modes: (1) Continuous Sending Mode and (2) Event Detection Mode. In the Continuous Sending Mode, the device sends the accelerometer data and the timestamp directly via the Bluetooth connection to a connected device. In the Event Detection Mode, the device is able to detect a special event, for example, a fall, and send the notification to a connected device. The Discovery Mini device communicates via Bluetooth 5.0 interface.

### 3.3. Radar Sensor Technology

#### 3.3.1. Why Radar Sensors?

The majority of sports are considered to be entertaining. However, some are dangerous for their participants. Consequently, some companies have developed tracking gadgets. Consider a player who is hit, resulting in one or more impacts. Then, a sensor (placed within the helmet, gloves, pants, neck guard, shirt, shin guards and elbow pads, hockey griddles, or other protective equipment) measures the impact and transmits the data to the coach and medical staff. This permits doctors to decide whether the player is fit to continue playing.

To maintain the patient’s well-being and early detection of conditions that may compromise their well-being, continuous remote monitoring of vital signs is essential (such as monitoring their breath level or heart rate) [29,30]. Medical Doppler radars are non-contact sensors that use the Doppler shift principle [30,31] to detect heartbeat and respiration rate by measuring the chest wall’s contraction and expansion [30,31]. This technology can also be applied to the sports field. By incorporating AI into the sports industry, we can avoid or forecast the onset of various diseases, making the sports field, which is a constantly fast-paced environment, a lot safer.

**Figure 2:**
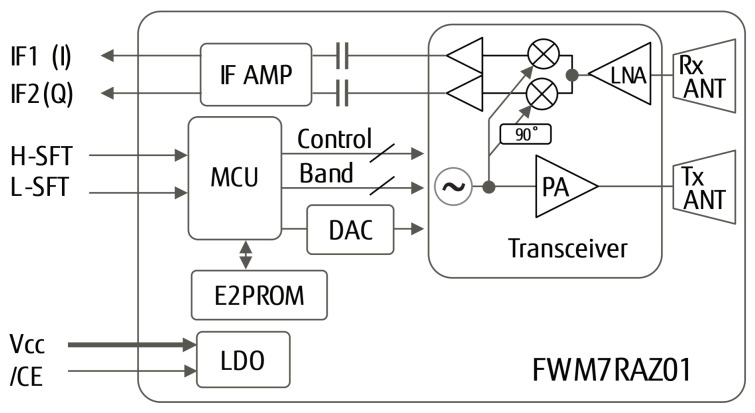
Functional block diagram of FWM7RAZ01 radar sensor [32,33].

Most of medical radar sensors presently on the market use Fujitsu’s radar module (FWM7RAZ01-200002) [32,33] which has 3 channels with dynamic selection and supports low-speed and high-speed object movements and varied but well-predefined data acquisition distances as well. The functional block diagram of FWM7RAZ01-200002 is shown on Figure 2.

**Figure 3:**
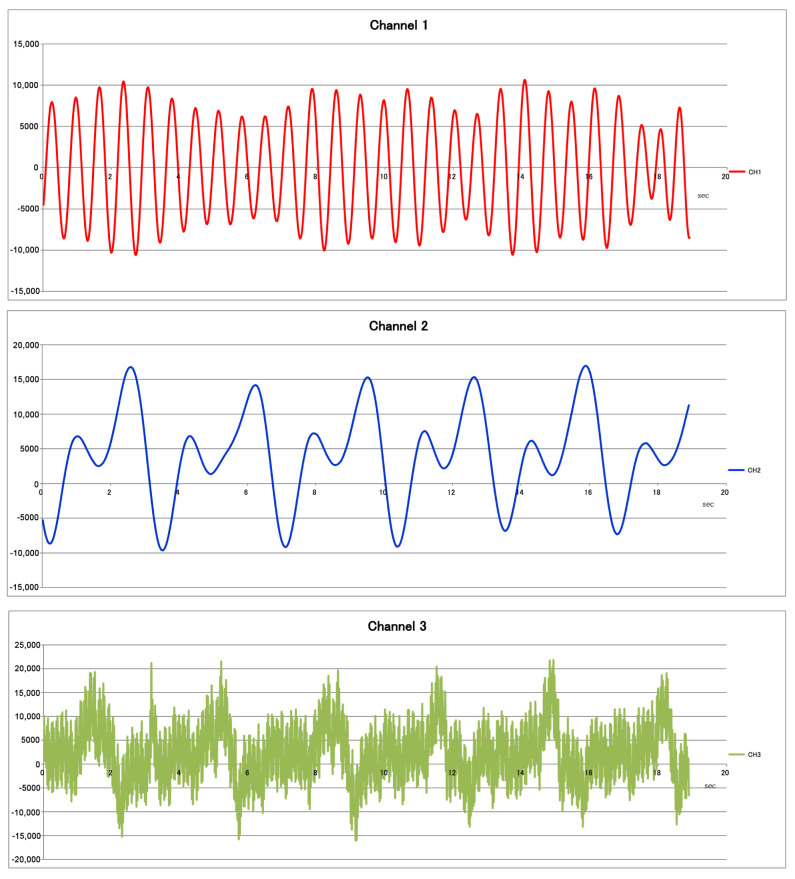
Channels of FWM7RAZ01 Doppler Radar Sensor.

This sensor is suitable for various applications, including vital sensing, machine monitoring, and other moving object detection and analysis. The following Figure 3 shows the radar’s three channels (Channel 1, Channel 2, Channel 3) with no blocking objects between the sensing device and the athlete. The FWM7RAZ01 Doppler radar sensor consists of a 24 GHz transceiver, TX and RX array antennas, IF amplifier, MCU, DAC, E2PROM, and an LDO voltage regulator all integrated into a single, compact module measuring 30 mm × 44 mm × 9.5 mm. Pin header terminals positioned on the back of the module simplify integration, even for designers who are not familiar with high-frequency circuits. Compared to other detection methods such as infrared, ultrasound, and laser scan, 24 GHz radio waves are more stable and less affected by environmental conditions, such as weather, temperature, illuminance, sound, or dirt. The waves also easily penetrate clothing, curtains, glass, and most wooden structures [32,33]. The calibration of the sensors needs to be conducted before starting every new type of sport or if we change the population per geolocation.

#### 3.3.2. Heart Rate and Rating of Perceived Exertion

By measuring the effect of a sports activity on a person’s heart rate and breathing rate, one can assess the intensity of the sports activity. There are two primary intensities of sports, as is shown in Table 2: moderate and vigorous intensity.

Borg implemented a scale to measure physical activity intensity called the RPE scale. Perceived exertion is a concept that describes how much effort a person believes their body is exerting on their behalf. It is based on the physiological sensations (e.g., increased heart rate, respiration, breathing rate, perspiration, as well as muscle tension) that an individual experiences while exercising [22]. These sensations are felt during physical activity. As a subjective measurement, it may be reasonable to assume that the exertion rating during physical activity on a scale from 6 to 20 provides a reasonably accurate indication of the heart rate during that activity, as Borg defined in 1998 [20].

The relationship between RPE and HR [21,22] has been studied several times in exercise physiology to achieve a better understanding. The concept of cognitive load is a way to determine whether a person believes they are working on a more complex task than their perceived output during physical activity. The amount of exertion is generally graded on a scale of 0 to 20, with 0 indicating the minimum exertion and 20 indicating the highest exertion.

Studies have demonstrated an association between HR and RPE, with a usual increase in HR matching an increase in RPE. However, the link is not always obvious and can be affected by an individual’s level of fitness, training status, and the activity performed. From this perspective, the influencing elements in performance sports are minimal. A highly trained individual may require a more significant HR to accomplish the same perceived exertion as a less trained individual. Additionally, the activity undertaken can affect the association between HR and RPE. Some studies have discovered a higher relationship between HR and RPE during steady-state activity (e.g., jogging or cycling at a constant pace) than during intermittent or interval exercise (e.g., high-intensity interval training).

Despite these limitations, HR and RPE continue to be regarded as legitimate and reliable markers of exercise intensity [34] and are frequently used for exercise prescription and evaluation. HR can be utilized as an objective indicator of activity intensity. In contrast, RPE is a subjective indicator of how difficult an individual thinks the activity is. RPE is also associated with various physiological markers such as blood lactate, ventilatory threshold, and blood pressure, among others, and could be a useful tool to match the intensity of exercise with the correct intensity zone, which can optimize training and prevent overreaching or overtraining.

There is a strong correlation between a person’s RPE [35] multiplied by ten and their actual HR during physical activity; therefore, a person’s effort rating may provide a reasonable estimate of their actual HR when engaged in physical activity. In other words, if an individual has a perceived effort of 12, then 10 times 12 equals 120 beats per minute. If the heart rate is at its maximum, there should be around 120 beats per minute, as the heart rate should be as high as possible [35].

Consideration has been given to the research [21,22] regarding the relationship between RPE and HR [21] utilizing the Borg RPE scale [20,21,22] from 6 to 20 with the regression equation: HR=8.88×RPE+38.2 (beats/minute) [21]. Consideration must be given to the deviation factor. HR will have a poorer connection with the rating of perceived exertion when athletes execute dynamic (intense) and static exercises [20,21]. Validation of physical activity intensity is derived from pulse or HR level during physical activity by monitoring the target zone.

#### 3.3.3. HR Targets for Vigorous-Intensity Physical Activity

The correlation algorithms can assess the relationship between HR and RPE rating [21]. To calculate this correlation, numerous strategies have been developed in the literature. One example is the algorithm which uses a modified version of Pearson’s correlation coefficient to calculate the correlation between HR and RPE.

This algorithm uses the following steps: (i) acquire the HR and RPE data from a physical activity dataset; (ii) represent the data on a scatter plot diagram (HR on axis *x* and RPE on axis *y*; (iii) determine the correlation coefficient (*r*) between HR and RPE using the Equation (1) shown below:

(1) $$r=\frac{n\sum xy-\sum x\sum y}{\sqrt{n\sum x^{2}-{\left(\sum x\right)}^{2}}\sqrt{n\sum y^{2}-{\left(\sum y\right)}^{2}}}\;,$$

where *n* is the number of data points, *x* is the HR, *y* is the RPE, ∑x is the sum of all the HR values, ∑y is the sum of all the RPE values, ∑xy is the sum of all the products of the HR and RPE values, $\sum x^{2}$ is the sum of the squares of the HR values, and $\sum y^{2}$ is the sum of the squares of the RPE values.

The correlation coefficient *r* is defined as follows in the relation between HR and RPE: (a) if r=1, then we have a perfect positive correlation; (b) if r=−1, then a perfect negative correlation is visible; (c) if r=0, this is the case of no correlation; (d) if 0<r<1, then we are talking about a positive correlation; and at last, (e) if −1<r<0, then we are talking about an existing negative correlation.

It is important to note that this is just one example of a correlation algorithm for HR and RPE. Depending on the specific context or research question, different approaches or variations can be applied. Additionally, this algorithm provides the correlation between both variables, but another approach, such as regression or machine learning algorithms, could give more insight and accurate predictions.

### 3.4. Acceleration Measurement

#### 3.4.1. Physical Activity Intensity

It is often categorized as light, moderate, and vigorous exercise based on the absolute amount of energy expended during physical exercise [36]. Multiples of MET express energy expenditure [37,38]. The MET [39] estimates the energy expended during a particular activity. When someone is at rest, they will typically burn approximately three milliliters of oxygen per kilogram of body weight per minute (mL/kg/min) during this period of time, which is the amount of oxygen their body emits under those conditions.

To calculate the MET by using a 3-axial accelerometer [16], it is first necessary to collect data from the accelerometer during the activity of interest. Such data typically include acceleration measurements in the *x*, *y*, and *z* axes. Next, the data need to be processed to extract relevant information about the activity. This can involve techniques such as filtering and feature extraction. For example, the root mean square (RMS) of the acceleration data in each axis may be calculated, which can provide an overall measure of the intensity of the movement. Once the data are processed, the use of a regression model is needed, or an equation to convert the data into an estimate of MET. Several existing models and equations can be used for this purpose. In our research, we reused the outcomes of one of our previous research projects [16]. Still, they typically require calibration using data collected from a sample of people performing the activity while measuring energy expenditure [37,38] using more direct methods, such as indirect calorimetry.

Depending upon the sport or activity one is participating in, the level of granularity or precision required, and the type of device and sensor that is being used, a different method might be employed. As explained in the paragraph above, the absolute level of aerobic exercise intensity is measured in terms of metabolic equivalents (METs).

A light-intensity activity requires less than 3.0 metabolic equivalents per minute for its performance to be effective, i.e., it is a non-sedentary waking activity. The amount of energy expended by walking at a speed of 2.0 miles per hour is 2.5 METs, so walking at 2.0 miles per hour is categorized as low-intensity exercise because the amount of energy expended is not more than 2.5 METs. There is a variety of light household tasks that need to be performed, such as cooking and light cleaning. METs are the power units that measure the intensity of an activity and are measured between 3.0 and 5.9. A walk at a speed of 3.0 miles per hour will require using 3.5 METs of energy before the activity is considered intense. This activity is therefore classified as moderate intensity because it involves moderate effort. Exercises such as walking at a fast speed, playing doubles tennis, raking the lawn, and playing tennis at a fast pace are some examples of aerobic exercises that can be performed. During a 1-mile run over 10 min at a speed of 6.0 mph, a metabolic rate of about 10 METs will be generated, which makes this an extremely vigorous activity. Exercise can take many forms, including running, jogging, lifting heavy objects, taking vigorous exercise classes, and even carrying heavy objects up and down the stairs [36].

To describe the amount of energy expended with a particular type of activity, METs or metabolic equivalents of tasks are valuable units of measurement. These units can be used to determine the amount of energy expended. A MET is defined as a ratio of the rate at which an individual spends energy when exercising to the rate at which they spend energy while at rest. For instance, the amount of energy a person consumes during rest can be estimated with the MET index. It has been estimated that if an individual engages in an activity for 30 min during which they perform 4 METs of physical activity, they will have completed 120 MET minutes of physical activity, equal to 2 MET hours of physical activity. To accumulate 120 MET minutes, one needs to perform 15 min of activities requiring 8 METs each at a pace of 2 METs per minute. This will enable one to accumulate 120 MET minutes [36].

#### 3.4.2. MET Calculation Algorithm

In order to calculate the metabolic equivalent of a task (MET), several algorithms have been proposed based on physical activity data, such as accelerometry or heart rate data. Depending on the nature of the activity and the data available, the specifics of the algorithm will vary, but in general, it will include the following steps:

1. Data collection: Collect data from the activity of interest using accelerometry, HR sensors, or other devices.
2. Data preprocessing: Clean and preprocess the data to remove any noise or outliers. This step may also include filtering and feature extraction to extract relevant information from the data.
3. Calibration: Establish a relationship between the physical activity data and energy expenditure [37,38] by using data collected from a sample of people performing the activity while also measuring energy expenditure by using more direct methods, such as indirect calorimetry.
4. Model development: Develop a model that can estimate MET from the physical activity data with the calibration data. This can be performed by using regression analysis or other statistical techniques.
5. Validation: Test the developed model with a separate dataset to ensure it is accurate and generalizable.
6. MET calculation: Use the developed model to estimate MET from new data.

It is crucial to highlight that the precision and accuracy of MET calculations will vary based on, among other things, the activity, the device and sensors used, and the participant sample. Different algorithms have been developed for various situations and sports, as well as for variable levels of granularity. Therefore, these factors are also crucial when deciding which algorithm to employ. The technique provided by Trost et al. in 2003, in their article “A Non-Exercise Prediction of Functional Aerobic Capacity” [40], is one example of a specific approach for calculating MET using accelerometry data. The algorithm used the following steps:

1. Collect accelerometry data from a sample of people performing different activities, such as walking, running, cycling, and resistance training.
2. Preprocess the data by removing any noise or outliers and calculating relevant features such as average acceleration, RMS, and variance of the acceleration data in each axis.
3. Calibrate the data by measuring energy expenditure in the same activities in a subset of the sample using indirect calorimetry.
4. Develop a regression model that relates the relevant features of the accelerometry data to energy expenditure using the calibration dataset.
5. Test the model on a validation dataset.
6. Use the developed model to estimate energy expenditure [37] in other new datasets.

The specific mathematical form of the regression model used in the algorithm is as follows:

MET = α + $\beta_{1}$(average acceleration) + $\beta_{2}$(RMS) + $\beta_{3}$(variance of acceleration in x-axis) + $\beta_{4}$(variance of acceleration in y-axis) + $\beta_{5}$(variance of acceleration in z-axis), where $\alpha ,\beta_{1},\beta_{2},\beta_{3},\beta_{4},\beta_{5}$ are the coefficients of the regression model, which are calculated from the calibration dataset.

The importance of the fact that this is only one example of a MET calculation algorithm needs to be highlighted. In addition to the type of accelerometer used to measure person’s acceleration, we need to take into consideration the activity being performed, and the characteristics of the sample of participants. Before using the algorithm to estimate MET in practice, it is crucial to validate the algorithm by analyzing data from a representative sample of individuals and activities. This will ensure that the algorithm has been adequately developed.

#### 3.4.3. Methods for Estimation Algorithm of Energy Expenditure

There are several different methods for estimating energy expenditure using medical radar sensors and 3-axial accelerometers. One common approach is to use the data from these sensors to estimate the individual’s metabolic rate, which measures the body’s energy at a given moment. This can be performed by using several algorithms based on physiological models and assumptions.

1. **Non-Parametric Approach**: One popular algorithm used for estimating energy expenditure is the Non-Parametric Approach to quantify the physical activity intensity (NPAI) algorithm. This algorithm takes raw data from the 3-axial accelerometer. It applies a series of mathematical transformations to convert the data into an estimate of the individual’s physical activity intensity (PAI) and energy expenditure.
  The NPAI algorithm integrates several data processing steps, including data cleaning, segmentation, feature extraction, and classification. The Non-Parametric Approach to Quantifying Physical Activity Intensity (NPAI) algorithm is a commonly used method for estimating energy expenditure from raw accelerometer data. The NPAI algorithm does not make any assumptions about the specific type of physical activity performed. It, therefore, can provide a comprehensive estimate of an individual’s overall physical activity level.
  The NPAI algorithm typically includes the following steps:
  Data cleansing is a process that involves removing artifacts or outliers from the accelerometer data that have been collected over the course of a period. During data segmentation, the accelerometer data are separated into segments corresponding to various activities, such as walking, running, and sitting, based on the data collected from the accelerometer. The third stage is the feature extraction stage. This involves extracting valuable elements from the accelerometer data, such as the mean value and standard deviation of the acceleration data. This way, different types of activity can be distinguished from each other. Based on the extracted features, segments are classified on the basis of their intensity of physical activity. Then, segments are classified on the intensity of physical activity of the extracted features. To estimate energy expenditure, data must first be segmented and classified. Based on the metabolic equivalent of each action (MET), the algorithm estimates the energy consumption associated with each action [41].
  Different ways to implement this algorithm include features and classification models being used to adapt it to different populations or scenarios. Additionally, the NPAI algorithm has been used to predict energy expenditure in several populations, including healthy adults and elderly individuals, and has shown a good correlation with reference methods. NPAI is a model-free method, which means it does not make assumptions about the physical activity being performed and the parameters of the body, which makes it useful in a wide range of scenarios and populations. Despite this, there may be limitations to the algorithm’s accuracy due to the quality of the accelerometer data and the specific implementation used in the algorithm. It is also essential to validate the algorithm with actual energy expenditure measurements to assess its performance.
2. **Doubly Constrained Energy Expenditure algorithm**: Another approach is the Doubly Constrained Energy Expenditure (DC) algorithm, which estimates energy expenditure by using both the 3-axis accelerometer and HR data. This algorithm assumes that HR and oxygen consumption are linearly related and that the oxygen consumption rate can be used to estimate energy expenditure. The DC algorithm uses a mathematical model to estimate the oxygen consumption rate from HR data. The accelerometer readings are then utilized to estimate the percentage of oxygen consumption attributable to physical activity. Combining data from 3-axial accelerometers and HR sensors, the DC method calculates energy expenditure. The algorithm presupposes that the HR is exactly proportional to oxygen consumption and that oxygen consumption can be used to predict energy expenditure.
  Typically, the DC algorithm consists of the following steps: (a) Data cleansing: at this stage, artifacts or outliers are removed from the accelerometer and HR data. (b) Estimating oxygen consumption: this phase involves applying a mathematical model to the HR data to determine the oxygen consumption rate; this model can utilize the Fick equation or other comparable models. (c) Estimating physical activity energy expenditure: at this stage, accelerometer data estimate the proportion of oxygen consumption attributable to physical activity; it is possible to link accelerometer data together with energy expenditure of physical activity by using a mathematical model to link the data. (d) Total energy expenditure estimation: the final phase estimates the individual’s overall energy expenditure by combining oxygen consumption and energy expenditure from physical activity.
  In addition, the accuracy of the algorithm may be constrained by the HR and accelerometer data quality and the algorithm’s implementation. As with any energy expenditure estimating method, it is essential to validate the DC algorithm using actual energy expenditure data to evaluate its performance.

Both algorithms are based on assumptions and models that may not be entirely accurate for all individuals, which is why validating the algorithm with actual energy expenditure measurements is essential. The methods should also be validated for different populations, such as elite athletes or patients with chronic diseases.

Another approach is to use machine learning (ML) techniques to develop models that predict energy expenditure from the sensor data. These models can be trained by using data from several individuals. They can be more robust and accurate than traditional algorithms, in particular when a good-quality dataset is used to train the model. However, similarly to traditional algorithms, these models also require validation.

## 4. Preprocessing

During preprocessing phase, we localized and selected the athletes from the relevant sports, practicing moderate and vigorous activities. After that, we set the minimal and maximal HR intervals to initiate further investigation processes. In the following Table 3, there is a predefined maximum HR per player with moderate and vigorous activity ranges. The details of calculations are listed in the upcoming Section 4.1 and Section 4.2.

### 4.1. HR Targets for Moderate-Intensity Physical Activity

Moderate-intensity physical activities are desired to be carried out at a heart rate (HR) between 64% and 76% of the maximum HR of the participant. A player can have a maximum HR of 220 beats per minute (bpm) minus their age. Here, HR ranges for moderate-intensity physical activities are listed:

1. Player 1, SubjectID 101 from PAMAP2: 27-year-old male athlete, Max HR 220 bpm-27 = 193 bpm, target HR between 124 bpm (Min. 64% = 193 bpm × 0.64) and 147 bpm (Max. 76% = 193 bpm × 0.76).
2. Player 2, SubjectID 102 from PAMAP2: 25-year-old female athlete, Max HR 220 bpm-25 = 195 bpm, target HR between 125 bpm (Min. 64% = 195 bpm × 0.64) and 148 bpm (Max. 76% = 195 bpm × 0.76).
3. Player 3, SubjectID 103 from PAMAP2: 31-year-old male athlete, Max HR 220 bpm-31 = 189 bpm, target HR between 121 bpm (Min. 64% = 189 bpm × 0.64) and 144 bpm (Max. 76% = 189 bpm × 0.76).
4. Player 4, SubjectID 104 from PAMAP2: 24-year-old male athlete, Max HR 220 bpm-24 = 196 bpm, target HR between 125 bpm (Min. 64% = 196 bpm × 0.64) and 149 bpm (Max. 76% = 196 bpm × 0.76).
5. Player 5, SubjectID 105 from PAMAP2: 26-year-old male athlete, Max HR 220 bpm-26 = 194 bpm, target HR between 124 bpm (Min. 64% = 194 bpm × 0.64) and 147 bpm (Max. 76% = 194 bpm × 0.76).
6. Player 6, SubjectID 106 from PAMAP2: 26-year-old male athlete, Max HR 220 bpm-26 = 194 bpm, target HR between 124 bpm (Min. 64% = 194 bpm × 0.64) and 147 bpm (Max. 76% = 194 bpm × 0.76).
7. Player 7, SubjectID 107 from PAMAP2: 23-year-old male athlete, Max HR 220 bpm-23 = 197 bpm, target HR between 126 bpm (Min. 64% = 197 bpm × 0.64) and 150 bpm (Max. 76% = 197 bpm × 0.76).
8. Player 8, SubjectID 108 from PAMAP2: 32-year-old male athlete, Max HR 220 bpm-32 = 188 bpm, target HR between 120 bpm (Min. 64% = 188 bpm × 0.64) and 143 bpm (Max. 76% = 188 bpm × 0.76).
9. Player 9, SubjectID 109 from PAMAP2: 31-year-old male athlete, Max HR 220 bpm-31 = 189 bpm, target HR between 121 bpm (Min. 64% = 189 bpm × 0.64) and 144 bpm (Max. 76% = 189 bpm × 0.76).

### 4.2. Heart Rate Targets for Vigorous-Intensity Physical Activity

The target HR for vigorous-intensity physical activities should be between 77% and 93% of the maximum HR of the participants. The maximum HR is 220 min the age of the player. Here, HR ranges for vigorous-intensity physical activities are listed:

1. Player 1, SubjectID 101 from PAMAP2: 27-year-old male athlete, Max HR 220 bpm-27 = 193 bpm, target HR between 149 bpm (Min. 77% = 193 bpm × 0.77) and 179 bpm (Max. 93% = 193 bpm × 0.93).
2. Player 2, SubjectID 102 from PAMAP2: 25-year-old female athlete, Max HR 220 bpm-25 = 195 bpm, target HR between 150 bpm (Min. 77% = 195 bpm × 0.77) and 181 bpm (Max. 93% = 195 bpm × 0.93).
3. Player 3, SubjectID 103 from PAMAP2: 31-year-old male athlete, Max HR 220 bpm-31 = 189 bpm, target HR between 146 bpm (Min. 77% = 189 bpm × 0.77) and 176 bpm (Max. 93% = 189 bpm × 0.93).
4. Player 4, SubjectID 104 from PAMAP2: 24-year-old male athlete, Max HR 220 bpm-24 = 196 bpm, target HR between 151 bpm (Min. 77% = 196 bpm × 0.77) and 182 bpm (Max. 93% = 196 bpm × 0.93).
5. Player 5, SubjectID 105 from PAMAP2: 26-year-old male athlete, Max HR 220 bpm-26 = 194 bpm, target HR between 149 bpm (Min. 77% = 194 bpm × 0.77) and 180 bpm (Max. 93% = 194 bpm × 0.93).
6. Player 6, SubjectID 106 from PAMAP2: 26-year-old male athlete, Max HR 220 bpm-26 = 194 bpm, target HR between 149 bpm (Min. 77% = 194 bpm × 0.77) and 180 bpm (Max. 93% = 194 bpm × 0.93).
7. Player 7, SubjectID 107 from PAMAP2: 23-year-old male athlete, Max HR 220 bpm-23 = 197 bpm, target HR between 152 bpm (Min. 77% = 197 bpm × 0.77) and 183 bpm (Max. 93% = 197 bpm × 0.93).
8. Player 8, SubjectID 108 from PAMAP2: 32-year-old male athlete, Max HR 220 bpm-32 = 188 bpm, target HR between 145 bpm (Min. 77% = 188 bpm × 0.77) and 175 bpm (Max. 93% = 188 bpm × 0.93).
9. Player 9, SubjectID 109 from PAMAP2: 31-year-old male athlete, Max HR 220 bpm-31 = 189 bpm, target HR between 146 bpm (Min. 77% = 189 bpm × 0.77) and 176 bpm (Max. 93% = 189 bpm × 0.93).

## 5. Modeling

The following ML experiments were conducted within this research: *Energy Expenditure from HR*: Algorithm: linear regression [42], Libraries: numpy, pandas, sklearn; *Physical activity intensity from HR—option 1*: Algorithm: logistic regression [43] with OLS, Libraries: numpy, pandas, sklearn; *Physical activity intensity from HR—option 2*: Algorithm: DecisionTreeClassifier [44], Libraries: numpy, pandas, sklearn; *Physical activity intensity from HR—option 3*: Algorithm: Lasso [45], Libraries: numpy, pandas, sklearn; *Level of the accuracy of predicted activity intens*: Algorithm: RandomForestClassifier [46] OST, Libraries: pandas, sklearn; *RPE from time-series HR dataset*: Algorithm: RandomForestRegressor [47], Libraries: numpy, pandas, sklearn.

## 6. Data Analysis

By monitoring the RPE over a lengthy period, we may adapt the training intensity, volume, and recovery tactics to reduce overtraining and injuries. By substituting RPE with session monitoring, a more advanced model can be set up (sRPE). This will allow for the improved management of the training load of athletes, ensuring that they are neither overworked nor undertrained, and hence lowering the risk of injury and assuring optimal performance. Moreover, combining METs with other variables (such as RPE and sRPE) can produce a sophisticated model for a thorough understanding of the training load and ACWR, thus optimizing training regimens and reducing the injury risk per session. By monitoring, the ACWR assists specialists in managing the progression of their workload, gradually raising the intensity and volume to overcome being overworked and fostering appropriate adaptation while decreasing injury risk. By combining these variables, we can gain a deeper knowledge of training loads, conditioning parameters and suitable training assets, fatigue levels, and injury risk factors. With ML models, this information enables the creation of tailored training programs, strengthens performance, and improves injury prevention techniques.

**Figure 4:**
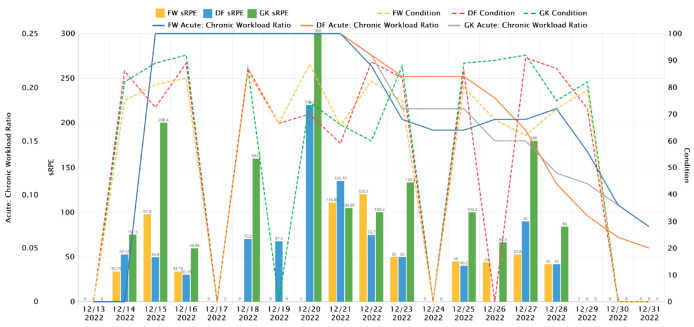
Daily sRPE, ACWR, Condition.

**Figure 5:**
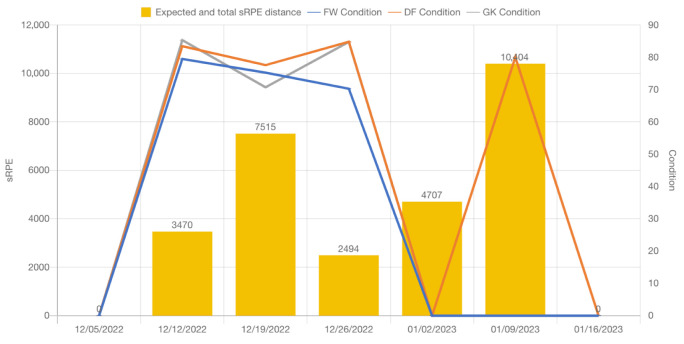
Weekly Expected sRPE, Condition.

In order to proceed with the METs, RPE, and sRPE, it is necessary to collect data during and after the activity sessions. In the case of numerous sessions per day, a more extensive analysis of sRPE can be prepared, as depicted in Figure 4 and Figure 5. These data points can be compiled by using radar sensor technology and 3D accelerometers, along with subjective reports from the athlete.

**Figure 6:**
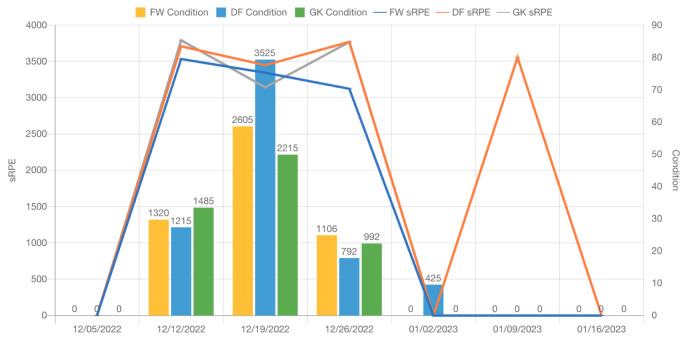
Weekly sRPE, ACWR, Condition.

Sports safety specialists and health professionals will benefit from manual and machine learning-assisted data analyses to identify trends and patterns that may be obvious or hidden, including changes in the heart rate, acceleration, and RPE/sRPE (see Figure 4, Figure 5 and Figure 6) score over time. Thanks to these data, one may be able to determine the general physical condition of the athlete, as well as their level of weariness and injury risk. It will be possible to identify any performance changes (Assessment of Fatigue and Injury Risk) or trends over time by comparing the estimated METs and RPE with the baseline data or historical data of the athlete. Therefore, it is possible for a training or rehabilitation plan to be developed in order to assist the athlete in reaching his or her goals. In order to minimize injuries and decrease weariness, this technique could be expanded as a “routinely monitoring asset” to track progress and identify any changes over time. This would ensure that athletes stay on track and accomplish their predetermined goals.

## 7. Results

As a result of the experiments, the ML models recognized the physical activity intensity and estimated the energy expenditure on a realistic level and were able to bring detailed information about the athletes’ health and physical condition and their expected progress. It was possible to gain objective RPE, sRPE, MET, and Condition data to control the physical state without focused activities (e.g., manual completion of sRPE forms). Leave-one-out (LOO) cross-validation (CV) as well as out-of-sample testing (OST) methods were used to evaluate the level of accuracy in activity intensity prediction. The detailed information about the results is shown in the following sections.

### 7.1. RPE, ACWR in Sports Using Subjective Data from Athletes

The following charts show the RPE and sRPE and the ACWR data of the athletes as a result of the manual data processing process. Using sRPE [23] instead of the classic analysis of RPE provides a superior monitoring method for the training load, taking into consideration the intensity of the training sessions as well.

This part of development has been implemented with the support of the Sunbears Cloud Campus [9] and Toyo University during the “sRPE/ACWR/Condition” research phase—to support student-athletes and coaches with up-to-date conditioning state data—with the involvement of sports specialists and coaches. Figure 4 shows the daily sRPE, ACWR, and Condition per player type in an ice hockey team.

Figure 6 shows the weekly sRPE, ACWR, and Condition per player type in an ice hockey team [9].

Figure 5 shows the expected total of Condition and sRPE per player type in an ice hockey team [9].

### 7.2. RPE, METs in Sports Using HR Data

**Figure 7:**
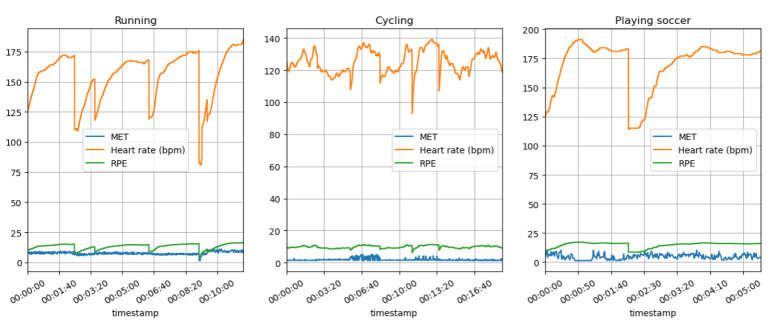
HR, MET, and RPE per sport.

The following Figure 7 shows the HR during running, cycling, and football (soccer) with predicted MET and RPE variations. The most significant metrics to consider when comparing these sports would be the METs level, which provides an objective measure of the energy expenditure, and the RPE values, which provide a subjective estimate of exertion, both critical measurements to take into consideration. As a result of these measures, tailored training can be designed, progress monitored, and sports safety key performance indexes (KPI) can be developed to prevent overtraining and/or injury among athletes.

**Figure 8:**
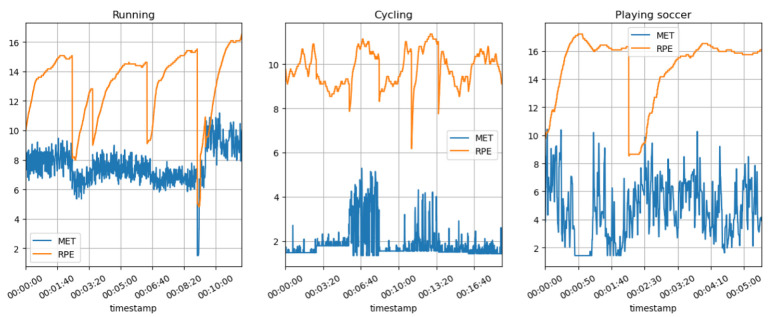
MET and RPE per sport.

Figure 8 shows the predicted MET and RPE variation during the running, cycling, and football (soccer) activities. The graph shows that the runners typically have a higher MET value than the cyclists and soccer players, indicating a higher level of energy expenditure and a greater amount of caloric expenditure in running. A higher rate of perceived exertion (RPE) may also be present during running, which indicates a greater degree of exertion during the activity. The MET value of cycling is generally lower than that of running, indicating a lower caloric expenditure as well as energy expenditure. While cycling may not be a cardiovascular workout as such, it can still be a beneficial one, especially when the intensity is moderate or vigorous, just as we experienced during our exercise. The RPE levels for cycling, according to the data, are likely to be lower than the RPE levels for running, indicating that cycling is a less intense activity than running. Football (soccer) differs from many other sports because it involves bursts of full-out exertion followed by short periods of rest. As a result, the MET values may be comparable or slightly higher than those of intensive cycling. The RPE levels are likely to be higher in the sport of running due to its intensity and the fact that it is stop-and-go throughout.

**Figure 9:**
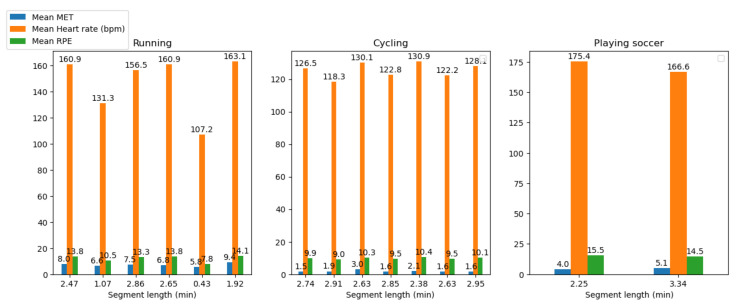
MET, HR, and RPE per sport on segments.

Figure 9 shows the HR with the predicted MET and RPE segments variations during the running, cycling, and football (soccer) activities.

**Figure 10:**
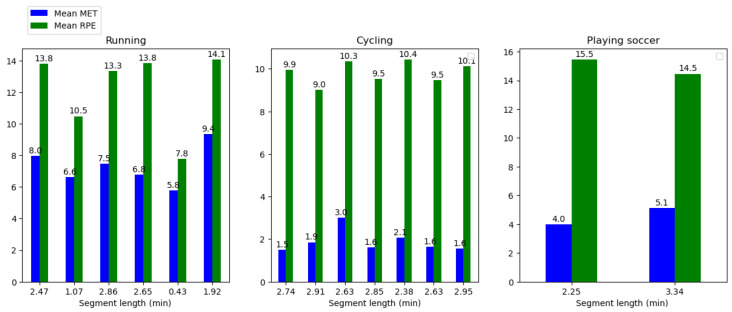
Predicted MET and RPE per sport on segments.

Figure 10 shows the predicted MET and RPE segment variations during the running, cycling, and football (soccer) activities.

### 7.3. RPE, METs in Sport Using 3-Axial Acceleration Data

Athletes must be monitored at predetermined intervals to find out why they are not providing a top performance, for instance, after every training session or every few weeks, in order to determine whether there is any reason for them not performing at their best level. Furthermore, it is important to make sure that the athlete’s subjective reports of comfort and pain have also changed over time. In order to detect any changes in these values on a regular basis, athletes are recommended to be checked on a frequent basis to detect any changes or anomalies as quickly as possible and to take preventative measures to avoid additional injury and fatigue. As part of regular monitoring, the athlete can also make sure that their goals and performance levels are achieved and that they are performing at their peak.

**Figure 11:**
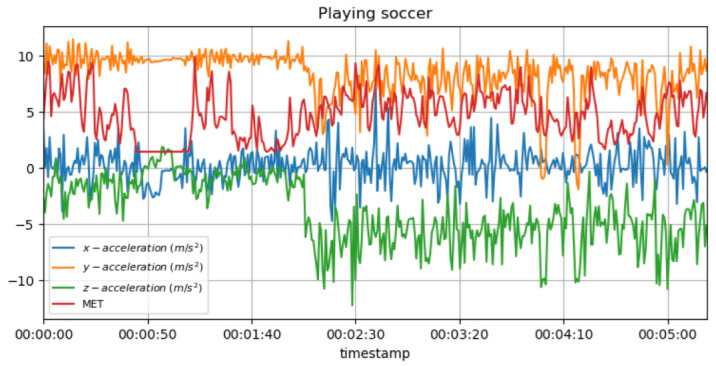
METs for football activity with acceleration data.

Because of the stop-and-go aspect of soccer, a player’s MET values may change at a specific point, providing evidence that discomfort is experienced in a specific location. If the individual’s MET values suddenly drop, it could also mean that the player is not able to perform at the same level of intensity, possibly due to an injury or fatigue, which may affect their individual ability to perform. Figure 11 shows the measured acceleration data on three axes with the predicted METs during the football (soccer) activity.

**Figure 12:**
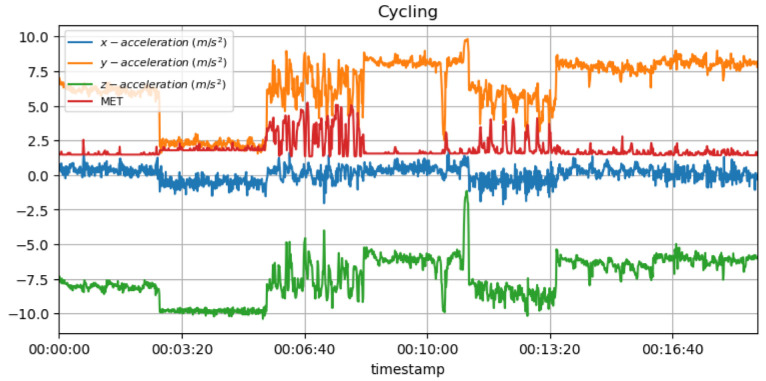
METs for cycling activity with acceleration data.

In the case of unexpected changes in the cyclist’s MET values during cycling, it may suggest that they are not capable of maintaining their normal intensity due to some injury or exhaustion, as a result of which they cannot perform their usual training. In addition, repetitive movements in cycling can place stress on certain joints, such as the knees, so it is important to monitor MET values on a regular basis in order to detect any overload issues that may arise in the future. Figure 12 shows the measured acceleration data on three axes with the predicted METs during the cycling activity.

**Figure 13:**
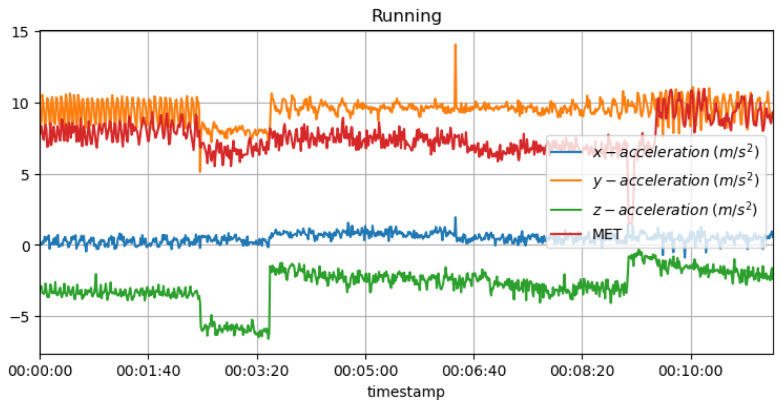
METs for running activity with acceleration data.

It is known that high MET values during strenuous jogging may suggest that the athlete is pushing too hard, thus increasing the risk of overtraining and injury as a result. In addition, a sudden drop in MET values may also indicate that the individual is not training as intensely as previously, possibly due to an injury or exhaustion, which may be the cause of the lowered MET values. Figure 13 shows the measured acceleration data on three axes with the predicted METs during the running activity.

In order to identify physical signs that may indicate injury or weariness, it is helpful to observe the training results as well as the joint’s range of motion. It is possible to identify hidden patterns or anomalies that may indicate injury or fatigue risk specific to an athlete through monitoring acceleration data. Suddenly changing acceleration patterns may indicate a muscle strain or joint injury, while a gradual decrease in acceleration may indicate fatigue or overload. Based on the rating of perceived exertion (RPE), we can determine the intensity of the workout as well as the level of fatigue. Increased RPE values may indicate that an athlete is exerting excessive effort and is at risk of injury or fatigue, in a similar way to acceleration data. In invisible, distant, or specific circumstances, wearable technology such as accelerometers and heart rate monitors can provide valuable information for a continuous progress assessment.

**Figure 14:**
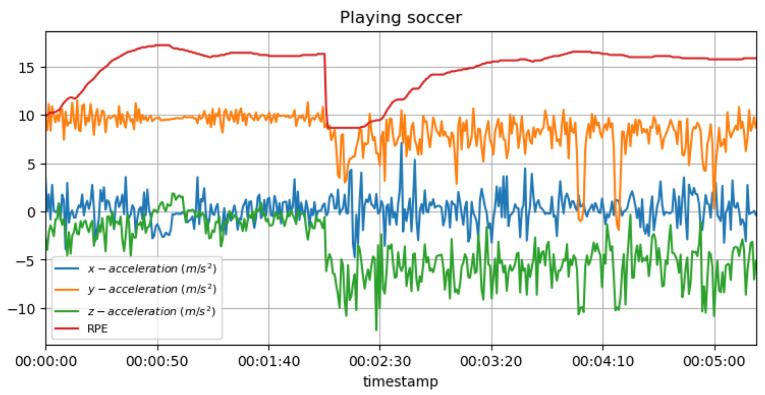
RPE for football activity with acceleration data.

Figure 14 shows the measured acceleration data on three axes with the predicted RPE during the football (soccer) activity.

**Figure 15:**
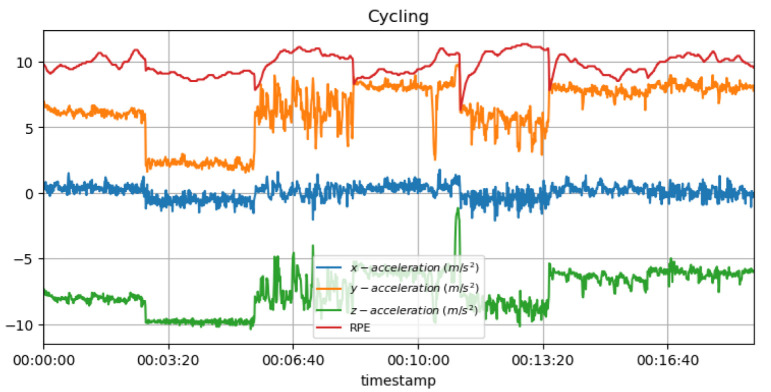
RPE for cycling activity with acceleration data.

Figure 15 shows the measured acceleration data on three axes with the predicted RPE during the cycling activity.

**Figure 16:**
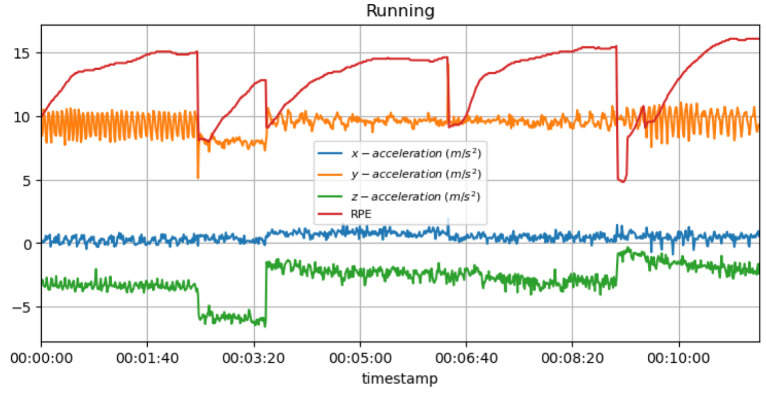
RPE for running activity with acceleration data.

Figure 16 shows the measured acceleration data on three axes with the predicted RPE during the running activity.

**Figure 17:**
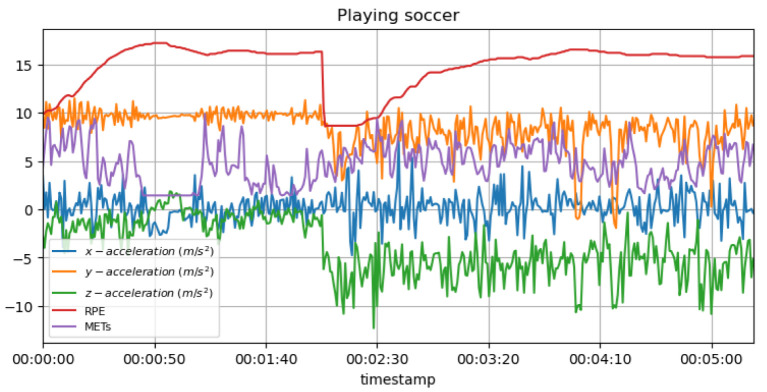
METs and RPE variations for football activity with acceleration data.

Figure 17 shows the measured acceleration data on three axes with the predicted RPE and METs variation during the football (soccer) activity. It is possible to suggest exhaustion or injury if the athlete’s heart rate is substantially lower than expected or if the acceleration data values fluctuate widely. An individual may experience discomfort or pain in a particular area if their heart rate or acceleration data values change rapidly.

**Figure 18:**
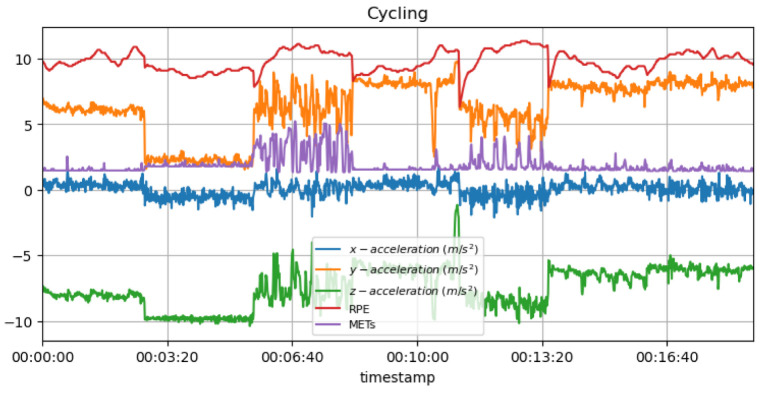
METs and RPE variations for cycling activity with acceleration data.

Figure 18 shows the measured acceleration data on three axes with the predicted RPE and METs variation during the cycling activity. An athlete with a low heart rate or whose acceleration data values vary considerably may be fatigued or get injured. Alternatively, a rapid change in heart rate or acceleration data may indicate discomfort or pain in a particular location.

**Figure 19:**
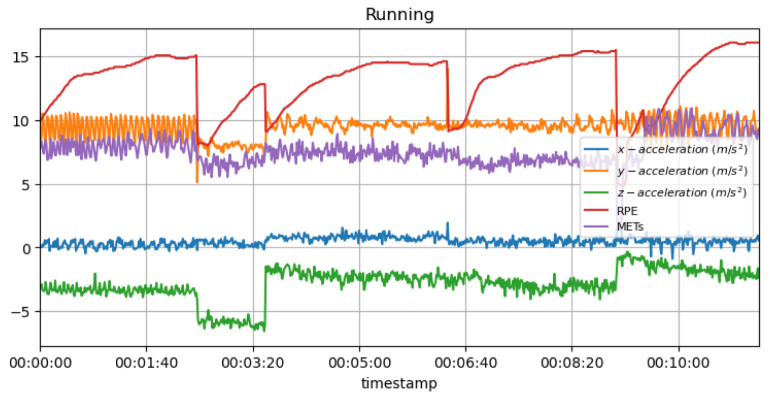
METs and RPE variations for running activity with acceleration data.

Figure 19 shows the measured acceleration data on three axes with the predicted RPE and METs variations during the running activity. A low heart rate or significant fluctuations in the acceleration data may indicate exhaustion or injury in an athlete. As well as indicating pain or discomfort, it may indicate that the individual is unable to maintain their normal level of intensity.

## 8. Discussion

As shown in Figure 4, the daily sRPE (Figure 4, Figure 5 and Figure 6 and Figure 10), ACWR (Figure 4 and Figure 6), and Condition (Figure 4, Figure 5 and Figure 6)—per player type in an ice hockey team—provide a complete picture of an individual’s level of physical activity and their progress toward achieving their short- and long-term goals (see Figure 5), as well as allow the development of training plans and sessions that are best suited to meet their needs, either at the group or individual level (Table 3). Additionally, they can assist in detecting when the individual may be overtraining, taking into consideration the HR intervals per activity type as we calculated in Section 4.1 and Section 4.2. They can monitor how well the individual is recovering and ensure that the individual does not overtrain, which can result in injury.

Changes in the MET values over time as well as unexpected declines or increases in these values are the most important KPIs for evaluating injury risk or anomalies. Additionally, a detailed subjective report from the individual about any discomfort or pain experienced by them can be valuable in identifying any potential injuries or anomalies. Regular monitoring of these KPIs can prevent damage and guarantee that the individual can perform at their top level.

### 8.1. Radar Sensors in Sports

Medical radar sensors (using the functional block diagram shown in Figure 2) relate to the application of radar technology in the medical, sports, and sport safety fields. This may involve using radars to measure physiological indicators, such as breathing and HR (see Figure 3). This technology can be utilized in hospitals, clinics, and even patients’ homes, but based on our experience, they need careful preparation to fit into the specific domain, taking into consideration the following: (1) The radar needs to avoid places with extreme weather conditions (e.g., intensive variations in humidity and temperature. (2) Before being used, it has to be calibrated for specific sports and athletes. (3) It needs to be installed in places where they can make (trigger-based) measurements of specific athletes. (4) If the athlete is changed, a dedicated solution for switching the users with predefined settings should be used. (5) Data acquisition requires a protocol that covers data acquisitions per multiple radar and multi-users. (6) In the case of sports, it is proposed that the medical radar sensor be installed on chairs or benches.

Radar-based physiological sensing (RPS) is an application of radar technology that measures the physiological aspects of the human body, such as HR (see Figure 3), breathing rate, and movement. One of the primary advantages of medical radar sensors is that they can monitor patients continually without needing physical touch or cameras. The field of medical radars is a relatively young discipline, and research is underway in this area. Nevertheless, several studies have already established the viability of utilizing radars to measure physiological indicators, and several businesses have begun developing commercial products based on this technique. The novelty of the RPS technology is that it is a non-contact method which is more comfortable and convenient for patients, offers a new way of continuous monitoring, and can be used in various scenarios, such as remote monitoring, home care, and ambulatory monitoring. Additionally, as the technology is still at an early stage, there is significant potential for future advancements and applications.

By installing medical radar sensors into benches and chairs used by athletes under the monitoring process, we will get free from transmitter factors or intentional interference datasets.

Some technical challenges still need to be overcome to make radar-based physiological sensing more accurate and reliable, such as interference from other sources and the effects of a patient’s movements on the radar signal. However, researchers are actively working on these issues and making progress. Based on our experiments, if the athletes do not realize that they are under a monitoring process, and we use installations close to them, as we mentioned before, then the quality of measurements will be higher. Radar sensors can be used as a source of HR data acquisition. The examination as a clinical trial was not the scope of this research. In this paper, we conducted experiments to measure and predict, using ML models, the RPE and sRPE (see Figure 7) and analyzed the context between MET and RPE (see Figure 8).

### 8.2. Limitatons of Medical Radar Sensor Technology

Medical radar sensors represent a relatively new technology that has the potential to revolutionize the way we measure and monitor vital signs.

1. The precision of medical radar sensors is one of their primary limitations. Although they can accurately monitor vital indicators such as HR, breathing rate level, and blood flow, their measurements can be affected by factors such as body posture, motion, and other objects within the field of view. In addition, the accuracy of the measurements may be influenced by the type and configuration of the radar sensor employed.
2. Another issue is their ability to permeate various materials and electronic gadgets. The signal of medical radar sensors, which typically operate at a few gigahertz, is absorbed or dispersed by elements such as water, metal, or any clothing worn by the patient.
3. Medical radar sensors are also sensitive to other electronic devices that operate at the same or similar frequencies, so the presence of cell phones, WiFi routers, or other medical equipment can interfere with their findings.

Despite these limitations, medical radar sensors are a reliable and valuable tool for monitoring vital signs in various medical applications, such as telemedicine, remote patient or player monitoring, and postoperative care.

### 8.3. Radar Sensors with Three-Axial Accelerometers in Sports

The combination of medical radar sensors with 3D accelerometers is a relatively new development in medical sensing. Using both types of sensors makes it possible to obtain more accurate and detailed information about a patient’s physiological status and physical activity. The accelerometer data can be used to estimate a person’s relative physical activity intensity (RPAI) (see Table 3) with METs, RPE, and sRPE, which can provide information about the activity level at which the athletes are performing at a given/expected moment (see sRPE in Figure 5). RPAI are a non-parametric approach to quantifying the physical activity intensity by using raw data from the accelerometer; it can be used to infer the energy expenditure from the body and track the physical activity of the individual objectively.

When combined with medical radar sensors, the accelerometer data can be used to validate and enhance the information obtained from the radar. For example, the radar can measure a patient’s HR and respiration rate while the accelerometer can measure their physical activity level [16,31]: (i) It allows for more accurate predictions of the RPAI. With the three-axial accelerometer data, it is possible to classify different types of physical activity and estimate their energy expenditure as well as the RPAI, which can be helpful in health promotion and clinical and research settings. (ii) It is a novel and promising approach to monitoring patients’ physiological status and physical activity; it can open up new opportunities for remote patient monitoring, home care, and ambulatory monitoring. However, more research is needed to validate these systems’ effectiveness and address the technical challenges of specifically targeted domains.

### 8.4. AI in Precise Detection and Result Generation

AI is capable of analyzing complex datasets (from multiple sources, such as wearable devices, training records, and physiological measurements) and identifying patterns, trends, and correlations that are difficult or impossible for humans to discern, learning and adapting as new data become available, which enables them to continuously refine their predictions and suggestions. Classic computations and models, in comparison, may require manual revisions and may not account for new information as efficiently. Furthermore, AI can represent complex, non-linear relationships between variables, which can be difficult to express by using conventional computations and models. This enables AI to deliver more comprehensive aspects that contribute to performance, weariness, and injury risk.

Another added value is that ML models could provide real-time data analyzing pipelines, delivering instantaneous (near real-time) feedback and recommendations to athletes and coaches. This flexibility permits prompt modifications to training plans, rehabilitation procedures, and injury prevention measures. With deep learning and reinforcement learning, the sports safety system built on this concept will anticipate outcomes with greater precision than conventional models and deliver individualized recommendations based on the unique qualities of each athlete (e.g., conditioning level, training history, and injury risk factors). These sophisticated strategies can account for the complexity and uncertainties inherent to sports performance and injury risk more accurately.

There are of course additional future AI possibilities that can be adapted to improve conditioning/training performance and the injury detection as well as ensure better fatigue management, as we can connect additional training or sensing health data sources. Here are some options that we suggest:

Performance optimization can be strengthened with (a) linear regression models to deepen the modeling of the relationships between an athlete’s performance, training load, recovery time, and nutrition; (b) a supervised learning algorithm, (e.g., Support Vector Machines) to provide a classification or to perform the regression tasks, helping to identify factors that contribute to an optimal performance; and (c) decision trees/random forests algorithms to identify the key variables that impact performance, and support coaches to prepare data-driven decisions for training program adjustments.

Injury prediction might be expanded with (a) logistic regression models to predict the likelihood of injury based on factors such as the training load, previous injuries, and biomechanical variables; (b) neural networks to model complex relationships between input variables and injury risk, offering more accurate predictions compared to traditional models; and (c) Gradient Boosting Machines to improve the accuracy of injury predictions by combining multiple weak learners (e.g., decision trees) into a single, strong learner, by adapting an ensemble learning technique.

Fatigue control and management have the following improvement possibilities: (a) a time-series analysis (e.g., ARIMA and LSTM) to analyze and forecast the athlete’s fatigue level trends, enabling coaches to adjust training programs; (b) a k-Nearest Neighbors (kNN) analysis for a comparison and similarity analysis of athletes or/and training sessions based on factors such as RPE, sRPE, or heart rate variability, providing insights into appropriate recovery strategies; and (c) reinforcement learning to provide the most SMART solution—a real-time pipeline aiming to maximize performance indexes while minimizing fatigue and injury risk parameters. This model will require of course the most comprehensive input datasets.

## 9. Conclusions

Estimating the energy expenditure by using medical radar sensors and three-axial (3D) accelerometers is challenging. Still, it is an important area of research with many potential applications in health and sports performance.

1. **Non-invasive and continuous monitoring**: By using medical radar sensors (Figure 3) and three-axial accelerometers, it is possible to non-invasively and continuously monitor an athlete’s physiological status and physical activity (Figure 17, Figure 18 and Figure 19) during training and competition. This can provide valuable information for coaches and trainers to optimize performance and prevent injury.
2. **Estimation of energy expenditure**: The combination of medical radar and three-axial accelerometer data allows for more accurate estimates of energy expenditure, which can help monitor an athlete’s condition level (e.g., while playing football, cycling, and running) and recovery status.
3. **Objective assessment of physical activity**: RPAI with METs (Figure 11, Figure 12 and Figure 13), RPE (Figure 14, Figure 15 and Figure 16), sRPE (Figure 4, Figure 5 and Figure 6), and ACWR (Figure 5 and Figure 6) can provide an objective assessment of an athlete’s physical activity, which can be more accurate than self-reported measures (Figure 4, Figure 5 and Figure 6).
4. **Training intensity control**: The RPAI (see Table 3) with METs, RPE, sRPE, and ACWR can be used to control the intensity of training sessions, ensuring that the athlete is not overtraining or undertraining.
5. **Injury prevention**: By continuously monitoring an athlete’s physiological status and physical activity (METs, RPE, and sRPE), medical radar sensors and three-axial accelerometers can provide early warning signs of potential injuries, allowing coaches and trainers to intervene and prevent injuries from occurring.
6. **Recovery monitoring**: By monitoring an athlete’s RPAI with METs, RPE, sRPE, and ACWR, in conjunction with other physiological parameters, it becomes possible to monitor an athlete’s recovery status after a training session or competition.
7. **Improving performance**: The continuous monitoring and objective assessment of physical activity (Figure 7, Figure 9 and Figure 17, Figure 18 and Figure 19) provided by RPAI can be useful for coaches and trainers to optimize an athlete’s training program and make data-driven decisions that lead to an improved performance.

By delivering a comprehensive image of an athlete’s physiological status and physical activity, the combination of medical radar sensors and 3D accelerometers can transform how we monitor and train athletes. Energy expenditure prediction with three-axial (3D) accelerometer sensors using “linear regression” provided 97–99% accuracy on the PAMAP2 model with selected sports, and the ML-based RPE results using medical radar sensors and time-series HR dataset had an accuracy level varying between 90–96% on different sports, but these results achieved a much better accuracy level than we expected at the beginning of the experiments. The expected level of accuracy was examined by using different models. The average accuracy for all the models (RPE and METs) and setups was higher than 90%. We received a lower percentage of accuracy when we analyzed the limitations of the medical radar sensors in extreme conditions to gain information about the limitations. The ML models that classify the RPE and METs perform consistently.

## Figures and Tables

**Table 1:** PAMAP2 table, with age expressed in years, height in cm, weight in kg, heart rates in bpm.

Subject ID	Sex	Age	Height	Weight	Resting HR	Max HR	Dominant Hand
Player1	Male	27	182	83	75	193	right
Player2	Female	25	169	78	74	195	right
Player3	Male	31	187	92	68	189	right
Player4	Male	24	194	95	58	196	right
Player5	Male	26	180	73	70	194	right
Player6	Male	26	183	69	60	194	right
Player7	Male	23	173	86	60	197	right
Player8	Male	32	179	87	66	188	left
Player9	Male	31	168	65	54	189	right

**Table 2:** Moderate and vigorous intensity examples.

Moderate Intensity	Vigorous Intensity
Nordic walking Tennis in double Gardening Water aerobics Waterpolo	Running Tennis Aerobic Cycling Ice hockey Basketball Futsal Football Rugby American Football

**Table 3:** Preprocessing of PAMAP2 data [25,26]: max HR, moderate and vigorous activity intervals.

			Moderate Activity Ranges		Vigorous Activity Ranges		
Subject ID	Sex	Age (Years)	MAX HR (bpm)	Min HR (bpm)	Max HR (bpm)	Min HR (bpm)	Max HR (bpm)
Player1	Male	27	193	124	147	149	179
Player2	Female	25	195	125	148	150	181
Player3	Male	31	189	121	144	146	176
Player4	Male	24	196	125	149	151	182
Player5	Male	26	194	124	147	149	180
Player6	Male	26	194	124	147	149	180
Player7	Male	23	197	126	150	152	183
Player8	Male	32	188	120	143	145	175
Player9	Male	31	189	121	144	146	176

## Data Availability

The dataset used in this experiment is known as the “PAMAP2 Dataset” and is freely available for academic research; there are no (legal or other) constraints on using the data for scientific purposes [25,26]. The data are available upon request. The figures, architecture, dataset, and results charts of this study can be found at https://doi.org/10.6084/m9.figshare.22012757.v1 (accessed on 16 March 2023).

## Abbreviations

The following abbreviations are used in this manuscript:

AI	Artificial intelligence
ML	Machine learning
3D Accelerometer	Three-axial accelerometer
PAMAP2	Sample database used by this research
LOO	Leave one out
CV	Cross-validation
OST	Out-of-sample testing
SDC	Sudden cardiac death
FWM7RAZ01	Fujitsu’s Doppler radar sensor
MCU	Microcontroller Unit
RPE	Rating of perceived exertion
MET	Metabolic equivalent of task
RMS	Root mean square
NPAI	Physical activity intensity
DC	Doubly constrained energy expenditure algorithm
RPS	Radar-based physiological sensing
FHL	Functional health literacy
RDF	Resource description framework
IoT	Internet of things
HIS	Head impact sensors
DPC	Dedicated private cloud
Triple H	Heart–head–heat concept
SSN	Semantic sensor network
EEE-CS	Institute of Electrical and Electronics Engineers—Computer Science
MRS	Medical radar sensor
3D	Tri-axial
RPA	Relative physical activity
HR	Heart rate
EE	Energy expenditure
